# Supplementary material for: Silver Nanoparticle–PEDOT:PSS Composites as Water-Processable Anodes: Correlation between the Synthetic Parameters and the Optical/Morphological Properties
Source: Polymers (Basel). 2023 Sep 6;15(18):3675. doi: 10.3390/polym15183675 (PMC10536234; doi:10.3390/polym15183675)
Supplement: Supplementary file 1 [file polymers-15-03675-s001.zip › polymers-2582318-supplementary.pdf]

Silver nanoparticles-PEDOT:PSS composites as water-processable anodes: correlation between the synthetic parameters and the optical/morphological properties

Stefania Zappia[1]*, Marina Alloisio[2]*, Julio Cesar Valdivia[3], Eduardo Arias[3], Ivana Moggio[3]*, Guido Scavia[1]*, Silvia Destri[1]

[1] Istituto di Scienze e Tecnologie Chimiche "Giulio Natta" (SCITEC), Consiglio Nazionale delle Ricerche (CNR), via Alfonso Corti 12, 20133 Milano (Italy)
[2] Dipartimento di Chimica e Chimica Industriale (DCCI), Università di Genova, Via Dodecaneso 31, 16146 Genova (Italy)
[3] Centro de Investigación en Química Aplicada (CIQA), Boulevard Enrique Reyna 140, 25294 Saltillo (México)
* Correspondence: S.Z. stefania.zappia@scitec.cnr.it; M.A. marina.alloisio@unige.it; I.M. ivana.moggio@ciqa.edu.mx; G.S. guido.scavia@scitec.cnr.it

Supporting information

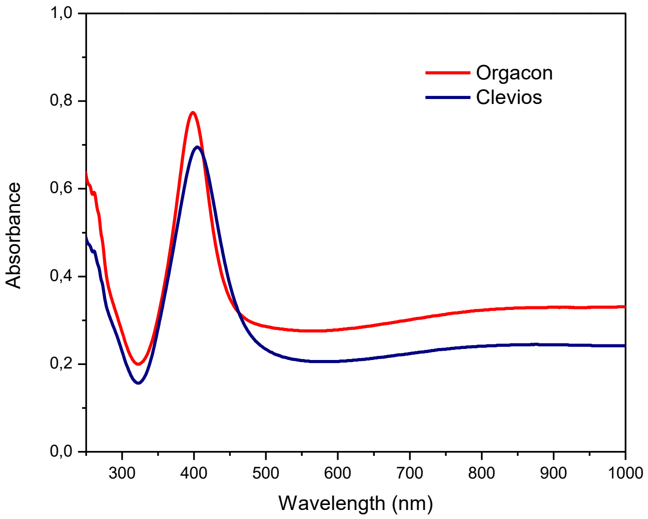

Figure S1. UV-Vis-NIR spectra of diluted starting suspensions: Orgacon (red line), Clevios (blue line).

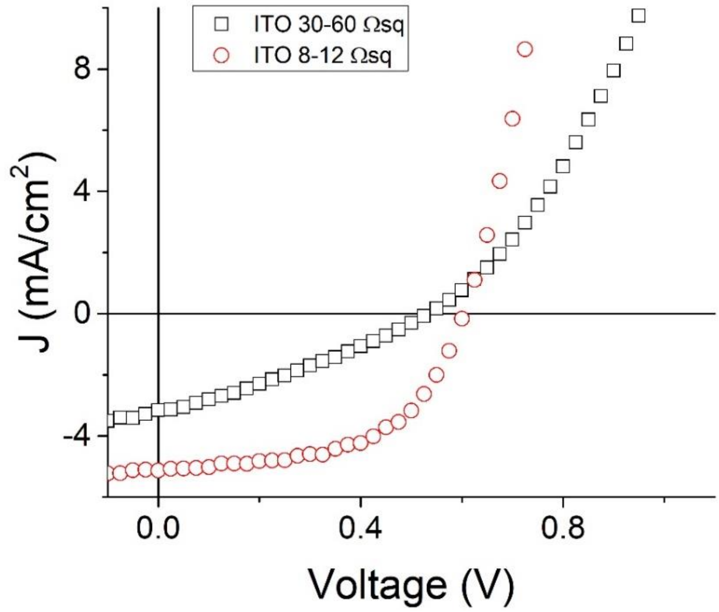

Figure S2. J-V curve for solar cells on ITO of different resistances.
